# Supplementary material for: Coupled Cluster Simulation of Impulsive Stimulated X-ray Raman Scattering
Source: J Phys Chem A. 2023 Oct 9;127(41):8676–84. doi: 10.1021/acs.jpca.3c03678 (PMC10591507; doi:10.1021/acs.jpca.3c03678)
Supplement: Supplementary file 2 — jp3c03678_si_002.pdf [file jp3c03678_si_002.pdf]

Supporting Information

Coupled Cluster Simulation of Impulsive Stimulated X-ray Raman Scattering

Alice Balbi,[1,*] Andreas S. Skeidsvoll,[2,*] and Henrik Koch[1,2,†]

[1]*Scuola Normale Superiore, Piazza dei Cavalieri, 7, I-56126, Pisa, Italy*

[2]*Department of Chemistry, Norwegian University of Science and Technology, 7491 Trondheim, Norway*

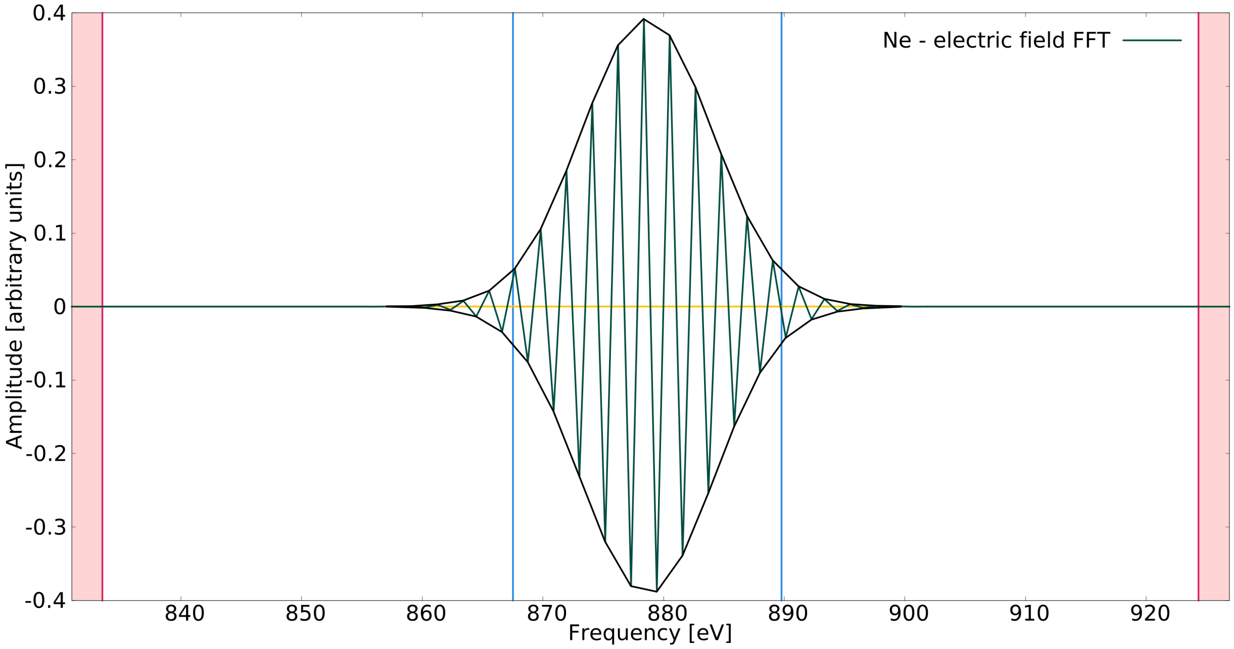

FIG. S1: Discrete Fourier Transform of the time-dependent electric field used in the simulation of impulsive stimulated x-ray Raman scattering (ISXRS) for neon at the CCS/aug-cc-pVDZ level. The blue vertical lines correspond to the calculated ground-to-core and valence-to-core excitations used for determining the carrier frequency of the pulse. In the areas with red background, the values of the discrete Fourier transform are on the order of 10^{-16} and lower.

* These authors contributed equally to this work.

† Electronic mail: henrik.koch@sns.it

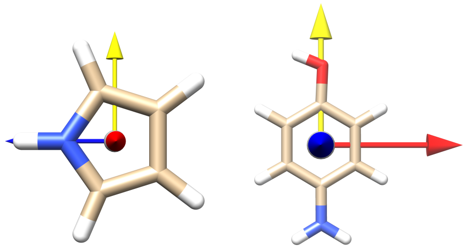

FIG. S2: Illustration of the geometry of pyrrole (left) and p-aminophenol (right) and the orientation of the molecules with respect to the chosen coordinate system: red arrow represents the x-axis, yellow arrow the y-axis, and blue arrow the z-axis.

TABLE S1: The carrier frequencies used for the Ne calculations, given in E_h, for different levels of theory and choices of basis set.

Basis set	CCS	CCSD
cc-pVDZ	32.4823669	32.108020
aug-cc-pVDZ	32.289568	31.734996
aug-cc-pCVDZ	32.287879	31.700736
aug-cc-pVTZ	32.272506	31.572652
aug-cc-pCVTZ	32.271458	31.585717
aug-cc-pVQZ	32.267882	31.557796
aug-cc-pCVQZ	32.267815	31.574858
aug-cc-pV5Z	32.266226	31.546955
aug-cc-pCV5Z	32.266177	31.566153
aug-cc-pV6Z	32.266359	31.546608

TABLE S2: The geometry used for pyrrole, in XYZ format.

N	0.0000	0.0000	1.1218
H	0.0000	0.0000	2.1178
C	0.0000	1.1209	0.3341
C	0.0000	-1.1209	0.3341
C	0.0000	0.7076	-0.9847
C	0.0000	-0.7076	-0.9847
H	0.0000	2.1084	0.7614
H	0.0000	-2.1084	0.7614
H	0.0000	1.3566	-1.8429
H	0.0000	-1.3566	-1.8429

TABLE S3: The geometry used for *p*-aminophenol, in XYZ format.

O	0.06378634	2.80129022	0.00000000
N	0.01292113	-2.79421806	0.00000000
C	0.00641697	-1.40619855	0.00000000
C	-1.19991305	-0.68608793	0.00000000
C	1.21060211	-0.67722621	0.00000000
C	0.00211501	1.42130088	0.00000000
C	-1.20111208	0.71186722	0.00000000
C	1.20931502	0.71706703	0.00000000
H	-2.15056588	-1.22171931	0.00000000
H	2.16273380	-1.21044479	0.00000000
H	-2.15295296	1.24754279	0.00000000
H	2.14745919	1.27120995	0.00000000
H	-0.84516387	-3.31799148	0.00000000
H	0.87658657	-3.30877159	0.00000000
H	-0.83159969	3.16001707	0.00000000
